# Solar Cell Applications of Solution-Processed AgInGaSe_2_ Thin Films and Improved Properties by Sodium Doping

**DOI:** 10.3390/nano10030547

**Published:** 2020-03-18

**Authors:** Xianfeng Zhang, Qingxuan Sun, Maoxi Zheng, Zhuohua Duan, Yuehui Wang

**Affiliations:** 1 Zhongshan Institute, University of Electronic Science and Technology of China, Zhongshan 528402, China; zhangxf07@gmail.com (X.Z.); zhmx1984@126.com (M.Z.); duanzhuohua@163.com (Z.D.); 2 Shenzhen Polytechnic, No. 7098, Liuxian Avenue, Nanshan District, Shenzhen 518000, China; 3 Guangdong Engineering-Technology Research Center of Nano-Photoelectric Functional Films and Devices, Zhongshan 528402, China

**Keywords:** AgInGaSe_2_ solar cell, non-vacuum method, sodium doping, conversion efficiency

## Abstract

Binary nanoparticle inks comprising Ag_2_Se, In_2_Se_3_, and Ga_2_Se_3_ were fabricated via a wet ball-milling method and were further used to fabricate AgInGaSe_2_ (AIGS) precursors by sequentially spraying the inks onto a Mo-coated substrate. AIGS precursors were annealed under a Se atmosphere for 1 h at 570 °C. Na_2_Se thin layers of varying thicknesses (0, 5, 10, and 20 nm) were vacuum-evaporated onto the Mo layer prior to the AIGS precursors being fabricated to investigate the influence on AIGS solar cells. Sodium plays a critical role in improving the material properties and performance of AIGS thin-film solar cells. The grain size of the AIGS films was significantly improved by sodium doping. Secondary ion mass spectroscopy illustrated slight surficial sodium segregation and heavy sodium segregation at the AIGS/Mo interface. Double-graded band profiles were observed in the AIGS films. With the increase in Na_2_Se thickness, the basic photovoltaic characteristics of the AIGS solar cells were significantly improved. The highest solar cell conversion efficiency of 6.6% (open-circuit voltage: 775.6 mV, short-circuit current: 15.5 mA/cm^2^, fill factor: 54.9%, area: 0.2 cm^2^) was obtained when the Na_2_Se thickness was 20 nm.

## 1. Introduction

The CuInGaSe_2_ (CIGS) thin-film solar cell is a very promising candidate for future large-area photovoltaic applications. The highest efficiency reported is 23.35% [1], which is comparable to that of monocrystal Si solar cells [2]. Further research of tandem solar cells based on CIGS solar cells is necessary to further increase solar cell efficiency. A wide bandgap of >1.70 eV has been calculated for the top cell as necessary to achieve a high efficiency of >30% when CIGS solar cells are used as the bottom layer [3]. AgInGaSe_2_ (AIGS) has been considered a suitable candidate as the tandem solar cell top layer. The highest efficiency of AIGS has been reported as 10.7% in our previous work [4]. Furthermore, the fabrication of AIGS absorber layers via a modified three-stage method using a molecular beam epitaxy (MBE) system has received a wealth of interdependent research [5,6]. However, the equipment of the MBE system requires a significantly elevated vacuum level and demands a high maintenance cost, which hinders large-scale applications in the photovoltaic industry. The non-vacuum method was widely used in fabricating CIGS [7,8] and Cu_2_ZnSnS_4_ thin films (CZTS) [9,10] because only simple equipment and fabrication process were required, which led to a low product cost. The conversion efficiency of non-vacuum fabricated CIGS and CZTS solar cells has respectively reached 17.1% [11] and 12.6% [12]. Herein, AIGS thin films were fabricated via a non-vacuum method to reduce the associated costs.

Sodium has been widely adopted to improve device performance and the material properties of CIGS-related materials [13,14,15]. Numerous reports have demonstrated the positive influence of sodium on the chalcopyrite absorber layer properties and solar cell performance. There are research efforts that have assigned the improvement associated with sodium on the chalcopyrite-related film grain size [16], while other reports have focused on the mechanism of how sodium doping influences the electronic and material properties [17]. Sodium is observed to passivate defects in CIGS-related films, resulting in improved solar cell performance [18,19,20].

**Figure 1:**
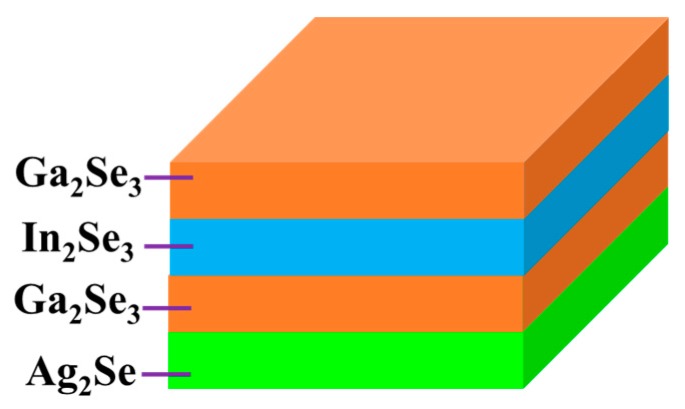
Structure of the AgInGaSe_2_ (AIGS) precursor.

In this work, a new non-vacuum method was adopted to fabricate low-cost AIGS absorber layers. Ag_2_Se, Ga_2_Se_3_, and In_2_Se_3_ nanoparticle inks fabricated via a wet ball-milling method were used to further fabricate AIGS substrate precursors. The AIGS precursor structure is shown in Figure 1. Such structure types ensure double-graded bandgap structures in the AIGS precursors, which are important for improving solar cell performance [21]. To study the influence of Na on the properties of AIGS films and solar cell performance, Na_2_Se layers of various thicknesses were vacuum-evaporated onto Mo-coated soda-lime glass (SLG) substrates prior to the deposition of the nanoparticle layers. The Na_2_Se thicknesses were selected as 0, 5, 10, and 20 nm. Thereafter, the AIGS precursor was annealed in a continuously pumped two-zone furnace under a Se atmosphere to improve the grain size, crystallization, and electronic properties. Se flux was continuously supplied to prevent decomposition of the AIGS film because of the low-Se atmosphere. The as-grown AIGS film was used as an absorber of the AIGS solar cell to complete the full solar cell structure. The solar cell performance was determined to evaluate the important role of sodium. The research herein provides a way to improve the properties of AIGS films and enhance the performance of AIGS photovoltaic devices.

## 2. Experimental Approach

### 2.1. Fabrication of Nanoparticle Inks

In this process, binary Ag_2_Se, In_2_Se_3_, and Ga_2_Se_3_ raw materials were provided by Kojundo Chemical Laboratory Co., Ltd (Sakado, Saitama Prefecture, Japan). And correspondent nanoparticle inks were fabricated via a wet ball-milling method, the procedures of which are explained in detail in our previous work [22]. During the milling process, 1 mm balls, 50 μm ceramic balls, one binary compound powder, and 5 mL ethanol were mixed together in a mill pot and milled for 40 h. Herein, three milling machines of the same model (P-4, Fritsch Japan Co., Ltd., Kanagawa Prefecture, Japan) were used to grind three different powders.

After ball milling, the mixtures were filtrated to obtain particles <32 μm. Through this procedure, all particles >32 μm were removed and a solution with relatively small particles (<32 μm) was obtained. Thereafter, 2-(2-ethoxyethoxy)ethanol and ethanol were used as a dispersion agent and solvent, respectively. The obtained solutions were then sonicated for 1 h to disperse the particles in the solvent. Next, the solutions were centrifuged twice: First, the centrifugation process was conducted at a low speed (1500 rpm) to remove particles over micrometers in size with the upper solution decanted; second, the centrifugation process was repeated three more times at a speed of 6000 rpm to gradually remove larger particles, to finally obtain the desired nanoparticles. Ag_2_Se, In_2_Se_3_, and Ga_2_Se_3_ nanoparticle inks having a concentration of 200 mg/mL were obtained by adjusting the quantity of ethanol. The obtained nanoparticle inks were then used to fabricate AIGS precursors using an inkjet printer. The Ag_2_Se layer was first deposited onto the substrate at a thickness of 0.9 μm, followed by sequential Ga_2_Se_3_ and In_2_Se_3_ layers having thicknesses of 0.7 and 0.25 μm, respectively. Finally, Ga_2_Se_3_ was again deposited onto the top layer to obtain a double-graded Ga distribution, which tailors the band profile of the AIGS films. The AIGS precursor structure is shown in Figure 1.

### 2.2. Sodium Doping Process

The back contact of the Mo layer, having a thickness of 800 nm, was deposited onto an ultrasonically cleaned SLG substrate. Na_2_Se layers of varying thicknesses (50, 100, and 20 nm) were fabricated onto the SLG/Mo substrates via a vacuum evaporation method, while a non-coated Na_2_Se layer-equivalent substrate was fabricated as a comparison.

### 2.3. Fabrication of AIGS Absorbers and Solar Cells

**Figure 2:**
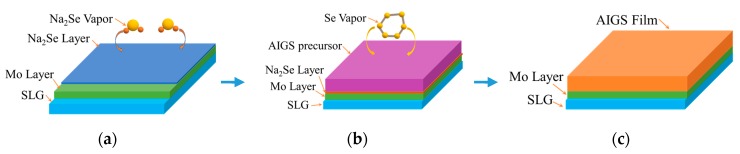
Schematic of the AIGS film fabrication process: (**a**) Na_2_Se fabrication process; (**b**) AIGS precursor annealing process; and (**c**) schematic of the AIGS film.

**Figure 3:**
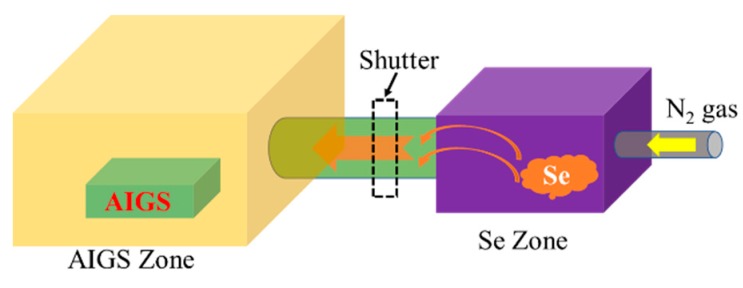
Schematic of the two-zone annealing furnace.

**Figure 4:**
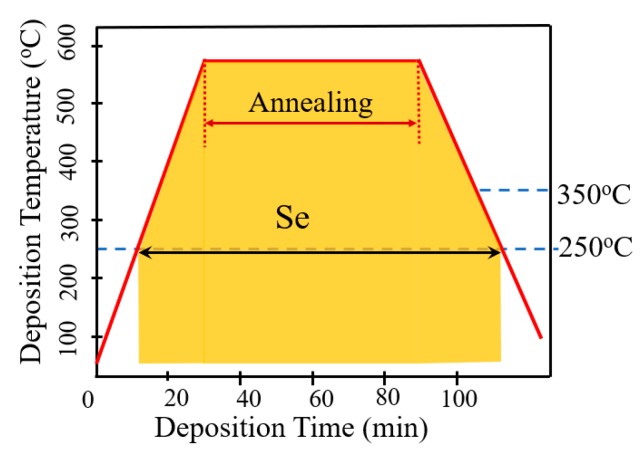
AIGS substrate temperature and Se flux profile during the annealing process.

Figure 2 schematically illustrates the AIGS film fabrication process. The AIGS precursor was fabricated using an inkjet printer. The Ag_2_Se, Ga_2_Se_3_, and In_2_Se_3_ nanoparticle inks were deposited sequentially based on the stoichiometric composition of the AIGS film. The design of the AIGS film structure, as shown in Figure 1, was to obtain AIGS films having a Ga/(In+Ga) ratio of ~0.75 to achieve a bandgap of ~1.70 eV. The precursors were thereafter annealed in a two-zone furnace, where the Se powder (99.999%) was placed in one zone and the AIGS precursor placed in the other zone. Figure 3 shows a schematic of the two-zone annealing furnace. A shutter between the two zones was used to control the Se flux in the AIGS zone. When the shutter was closed, Se is prevented from flowing to the AIGS zone, and only when the shutter is open does the Se flux flow to the AIGS zone. Prior to annealing, the shutter was left in the open position and both zones of the furnace were evacuated to ~2.0 × 10^−5^ Pa, using a molecular pump to remove oxygen. Thereafter, the shutter between the two zones was set to the closed position, and the substrate and Se sources were heated. The Se source was first heated to 150 °C in 40 min, to obtain a high Se flux prior to heating the AIGS substrate. The temperatures of the AIGS substrate and Se flux profile are shown in Figure 4. The substrate was heated to 570 °C in 30 min, with the thermocouple connected to the substrate to ensure a consistent and accurate temperature. When the substrate temperature was increased to 250 °C, the shutter between the two zones was opened in the presence of a N_2_ carrier gas to carry Se to the AIGS zone. The temperature was isothermally maintained at 570 °C for 1 h throughout the annealing process. Next, the substrate temperature was decreased to room temperature at a rate of ~7.5 °C/min, and only when the substrate temperature decreased to 350 °C was the shutter closed to stop the supply of Se flux. Finally, the as-grown AIGS films were removed from the annealing furnace and immediately characterized. During the annealing process, the molecular pump was shut down and the annealing furnace was continuously pumped using a mechanical pump to maintain an annealing pressure of 10 Pa, aiming at providing sufficient Se flux to promote selenization of the AIGS films.

A typical AIGS solar cell structure can be described as Mo/AIGS/CdS/i-Zno/ZnO:Al/Al. In this work, a 50 nm thick CdS buffer layer was deposited onto the as-grown AIGS films by a chemical bath deposition method, followed by a sputter-deposited i-ZnO (80 nm) layer and an Al-doped ZnO (600 nm) layer. Finally, a front-contact Al grid was deposited onto the top layer by an evaporation method.

### 2.4. Characterization

The morphology of the annealed AIGS films was characterized using a scanning electron microscope (JSM-7001F, JEOL, Tokyo, Japan) equipped with a JED-2300T energy-dispersive spectroscopy (EDS) system operating at an acceleration voltage of 10 kV. EDS for compositional analysis was measured at an acceleration voltage of 15 kV. The particle size of the inks was measured by transmission electron microscopy (TEM, JEM-2100F, JEOL, Tokyo, Japan). Crystallization of the AIGS films was characterized by X-ray diffraction (XRD, SmartLab, Rigaku, Tokyo, Japan) with a 40 kV voltage and 20 mA current. The elemental depth profile of the AIGS film was measured by secondary ion mass spectroscopy (SIMS, TOF.SIMS5, Hitachi, Tokyo, Japan), using a 3 keV primary Cs^+^ ion beam. The strong signal of the Mo back electrode was used as a characterization marker of the AIGS film rear surface. The solar cell performance was measured with a 913 CV type current–voltage (J–V) tester (AM1.5), provided by an solar simulator (LP-50B, EKO, Tokyo, Japan). The simulator was calibrated with a standard GaAs solar cell to obtain the standard illumination density (100 mW/cm^2^). The external quantum efficiency (EQE) of the AIGS solar cells was characterized by a QE tester (QE-2000, Otsuka Electronics Co., Ltd., Oosaka, Japan).

## 3. Results and Discussion

### 3.1. Nanoparticle Ink Characterization

**Figure 5:**
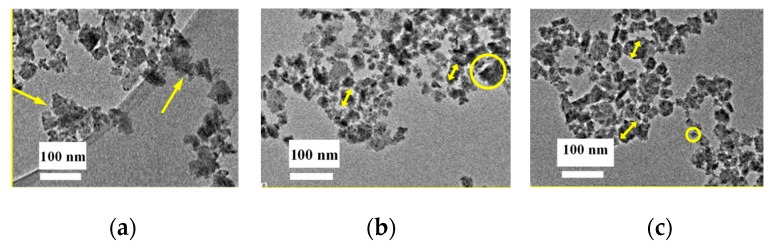
Transmission electron microscopy images of the (**a**) Ag_2_Se, (**b**) Ga_2_Se_3_, and (**c**) In_2_Se_3_ nanoparticle inks.

To perform the measurements, the nanoparticle inks were diluted 20 times to reduce the concentration of the particles in the ink. Additionally, the inks were sonicated for 1 h prior to being dipped onto the TEM sample holder micro-grid to reduce particle agglomeration. Figure 5a–c shows the TEM micrographs of the nanoparticle distributions in the inks of Ag_2_Se, In_2_Se_3_, and Ga_2_Se_3_, respectively. As shown in Figure 5a, the Ag_2_Se particle size varies from several nm to ~10 nm. Although the particle size distribution is broad, there are almost no observed particles >10 nm. The yellow arrows in the figure illustrate particle clusters. The cluster size is ~10 nm; unfortunately, specific particle sizes are difficult to identify because of the vague boundary. Judging from Figure 5b, the Ga_2_Se_3_ particle size distribution is relatively uniform and no significant agglomerations are observed. The particles exhibit a regular size of several tens of nanometers, as illustrated by the yellow arrows. Large particles of ~10 nm can also be observed, as shown in the circle, at a significantly lesser degree, however, when compared to the Ag_2_Se ink. The average particle size of In_2_Se, Figure 5c, is <10 nm, with the smallest particles having a size observed to be ~10 nm, as illustrated by the circle, while the largest particle is observed to be ~50 nm, as shown by the arrows. Thus, the wet-milling process is concluded to be efficient and the nanoparticle inks are obtained for all three materials.

### 3.2. Characterization of AIGS Films 

**Figure 6:**
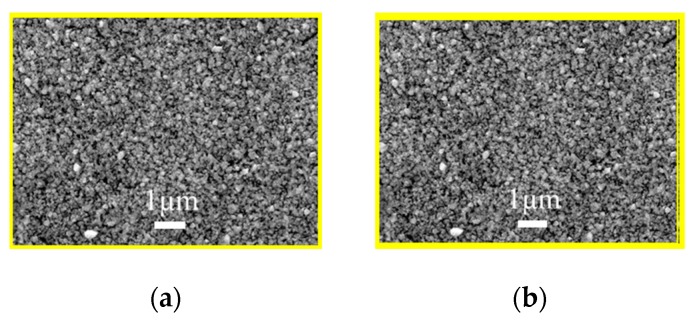
(**a**) Scanning electron microscopy surface morphology of the (**a**) AIGS precursor, and (**b**) cross-sectional micrograph of the AIGS precursor.

The as-milled nanoparticle inks were used to fabricate the AIGS precursors having a structure shown in Figure 1. The morphology of the AIGS precursor, as shown in Figure 6a, exhibits a compact and flat surface with no observable defects, such as pin holes or large surficial fluctuations. However, the specific grain size could not be measured, because of the unobvious grain boundaries. Judging from the cross-sectional image of the AIGS precursor, as shown in Figure 6b, the AIGS film shows a compact structure along the thickness direction. The particle size can be clearly measured. Although the precursor is composed of three layers, only a single layer of film can be observed as a result of the similarity of particle size for all the nanoparticle inks. To improve the crystallinity, grain size, and elemental distribution of the AIGS films, high-temperature annealing (570 °C) of the AIGS precursors under a Se atmosphere was conducted. Details of the annealing process are described in the experimental section. Furthermore, the samples were immediately characterized after being removed from the annealing chamber.

**Figure 7:**
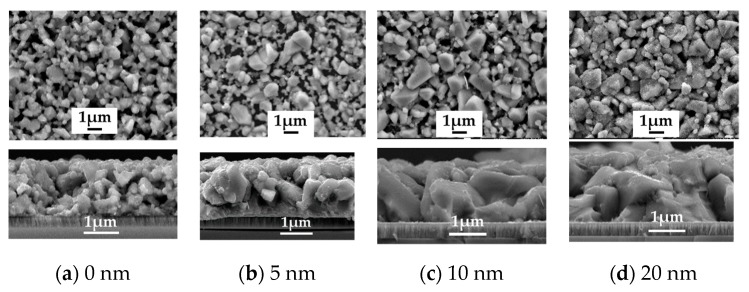
Surface and morphology of the AIGS films showing the important role of the Na_2_Se post-deposition process. (**a**) No Na_2_Se post-deposition. Na_2_Se thicknesses of (**b**) 5, (**c**) 10, and (**d**) 20 nm.

To study the role of Na_2_Se on the AIGS film and solar cell properties, a series of experiments were conducted by post-depositing various Na_2_Se layer thicknesses of 5, 10, and 20 nm. A film without Na_2_Se post-deposition was used as a reference. The improvement in the AIGS film grain size in the presence of Na is clear based on the observations from Figure 7a–d. For the AIGS precursor in the absence of any post-deposited Na_2_Se (see Figure 7a), the AIGS film exhibits a lesser degree of quality having a broad grain size distribution, where the grain size of the smaller particles is <20 nm, with the larger grain sizes being >1μm. By applying the Na_2_Se post-deposition prior to fabricating the AIGS precursors, the grain size was significantly increased with a reduction in surface roughness. The grain size gradually increased as a function of Na_2_Se thickness. Tailoring the thickness of Na_2_Se to 5 nm initiates growth of the grain size, where a maximum value is observed with a Na_2_Se thickness of 10 nm. Additionally, some of the grains extended throughout the film, with some of the smaller grains being observed at the bottom of the AIGS precursor. When the thickness of Na_2_Se increased to 20 nm, the grain size uniformity decreased significantly. Liquid Na_2_Se has been reported to play a critical role in triggering the mechanism during grain growth [23]. Furthermore, other research has demonstrated Se to be chemically adsorbed onto Na to form polyselenide Na_2_Se_x_ during the annealing process, which promotes the crystallization of the AIGS film because of the reaction between the metal precursor and reactive Se derived from Na_2_Se_x_ [24].

**Figure 8:**
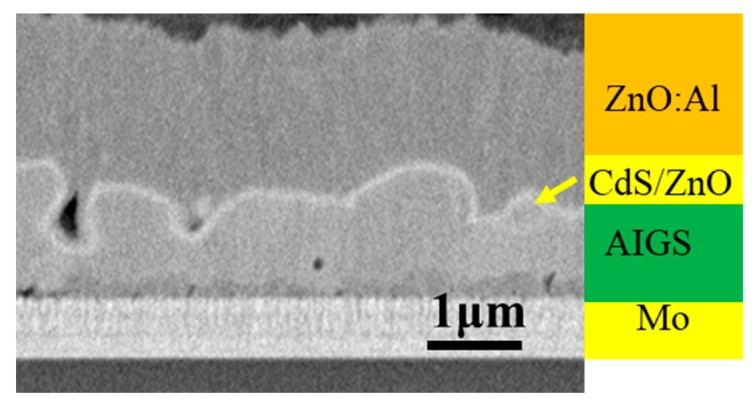
Cross-sectional SEM image of a typical AIGS solar cell.

**Figure 9:**
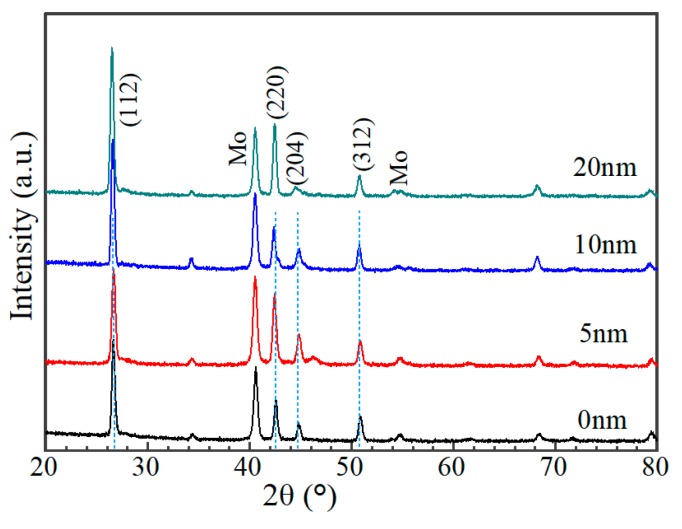
XRD patterns of AIGS films with different thicknesses of Na_2_Se.

Figure 8 shows the cross-sectional morphology of a typical AIGS solar cell. Pinholes and a severe rough surface can be observed. Figure 9 shows XRD patterns of AIGS films with different thicknesses of Na_2_Se layers. Main peaks of the chalcopyrite AIGS films are illustrated by blue lines. It is concluded that all the AIGS films show a typical chalcopyrite structure. It can also be observed that the peak intensity of AIGS films gradually increase with the increase in Na_2_Se thickness, indicating a better crystallinity. 

**Figure 10:**
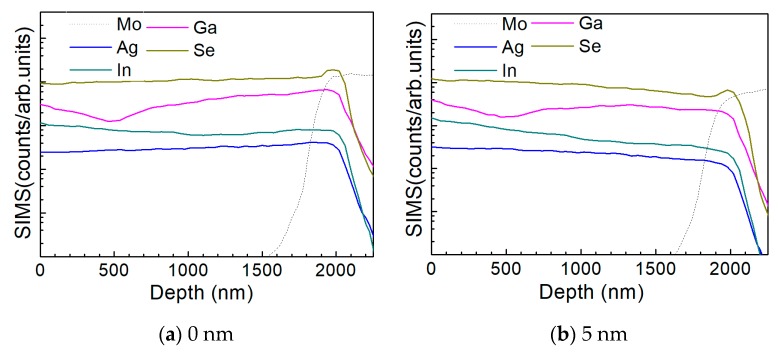

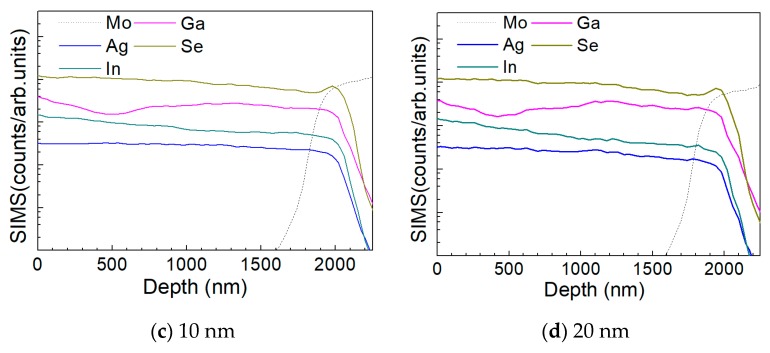
Elemental distribution profiles of AIGS films as a function of Na_2_Se thickness by secondary ion mass spectroscopy (SIMS): (**a**) 0, (**b**) 5, (**c**) 10, and (**d**) 20 nm.

Figure 10 a–d show the depth profile results of the element distribution of the AIGS solar cells as a function of Na_2_Se thickness (0, 5, 10, and 20 nm) by the SIMS method. All the samples were fabricated on SLG substrates. The sample in the absence of any post-deposition of Na_2_Se was used as a reference. Sodium was not added to the film intentionally, to have all the Na derived from the SLG substrate. The free surface of the AIGS film was set as the starting point of the SIMS measurement. The dotted line shows the elemental profile of Mo, and the rapid onset of the curve was used to identify the bottom of the AIGS absorber. The Ag, In, Ga, and Se elements in all the four samples show similar distribution profiles having small fluctuations throughout the absorber layer. An obvious ‘hump’ structure near the surface of the Mo layer in the Se profile was observed, indicating segregation of Se, which is attributed to the formation of a MoSe_2_ layer that is commonly observed in chalcopyrite solar cells [25]. Furthermore, the Ga distribution profile shows a ‘valley’ structure having a minimum concentration at ~500 nm beneath the surface of the AIGS film. As the AIGS film band profile is influenced by Ga content [26], a double-graded bandgap is inferred, which is beneficial for photon absorption [27,28], and thus, promotes solar cell performance.

**Figure 11:**
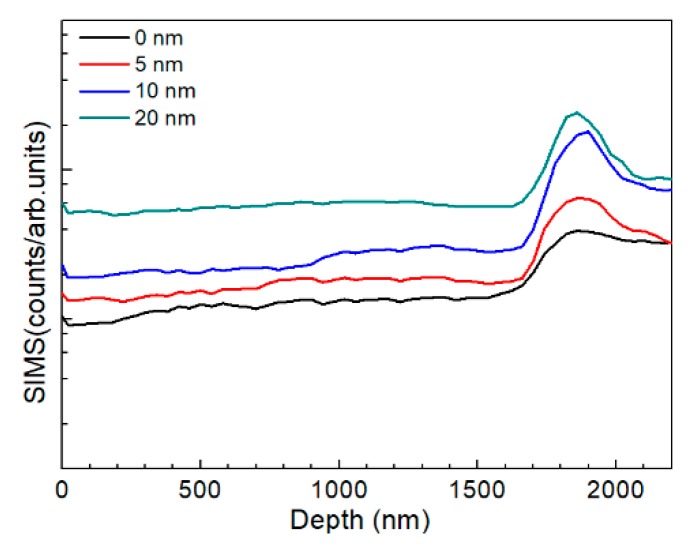
SIMS sodium profile as a function of Na_2_Se thickness.

The AIGS films were also characterized by SIMS to investigate Na distribution. Figure 11 shows the AIGS film sodium profile as a function of Na_2_Se thickness. For the AIGS film without any post-deposited Na_2_Se, the sodium element was observed to diffuse from the SLG substrate. Thus, the sodium concentration gradually increased from the front surface to rear surface. However, a slight intensity decrease in the profile was observed by focusing on the top surface of the film, indicating slight sodium segregation at the top surface. An obvious sodium segregation near the AIGS/Mo interface was observed, which is widely reported in CIGS solar cells [29,30]. For the AIGS film having the 5 nm Na_2_Se post-deposition layer, sodium diffusion to the surface was significantly promoted, with the sodium concentration increased throughout the film. With increased Na_2_Se thickness, the sodium concentration level in the AIGS film gradually increased with a concomitant increased segregation of sodium at the AIGS/Mo interface.

**Figure 12:**
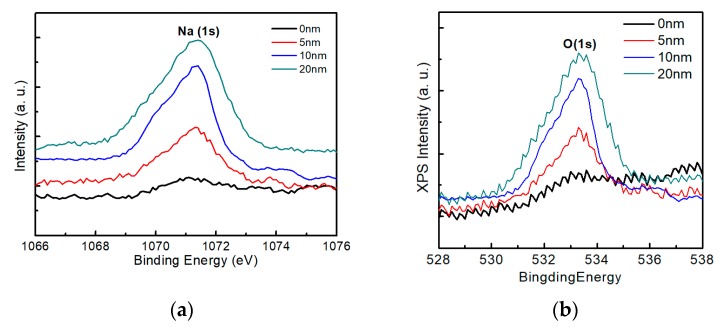
X-ray photoelectron spectroscopy measurements of samples containing various sodium contents: (**a**) Na spectra and (**b**) oxygen spectra.

Figure 12 shows the X-ray photoelectron spectroscopy spectra of sodium and oxygen on the surface of the AIGS films as a function of Na_2_Se post-deposition thickness. As the thickness of sodium increased, an obvious Na (*1s*) peak was observed, Figure 12a, which is consistent with the previous SIMS observations of surface sodium segregation. Additionally, the Na (*1s*) peak was accompanied by an O (*1s*) peak, Figure 12b, with a synchronous change in the intensity of the spectra. During the experiment, the samples were exposed to air for <5 min. These results indicate that oxygen was absorbed onto the AIGS surface within minutes for samples comprising high surface sodium levels, while the absorption of oxygen was low for samples with lower surface sodium levels. The relationship between sodium and oxygen has previously been reported in the case of CZTS [31], and a similar conclusion was obtained in this work.

### 3.3. Properties of AIGS Solar Cells 

**Figure 13:**
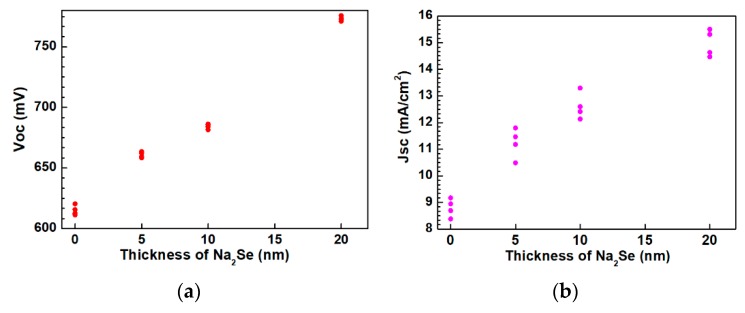

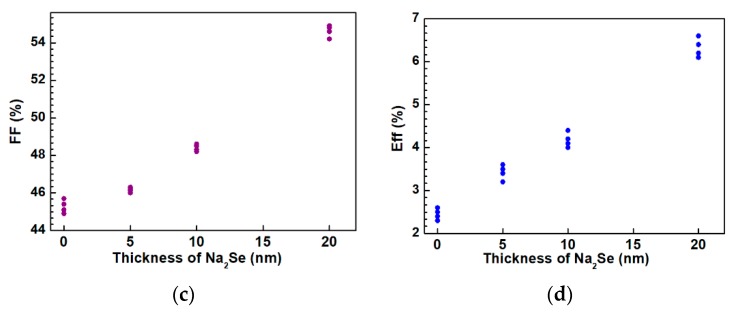
Photovoltaic parameters of solar cells as a function of Na_2_Se thickness: (**a**) Open-circuit voltage, (**b**) short-circuit current, (**c**) fill factor, and (**d**) conversion efficiency.

The as-grown AIGS films were used to complete the full structure of the solar cells. The AIGS solar cell performance was characterized under a standard condition of 100 mW/cm^2^ irradiation for illumination. A high-precision monocrystalline Si solar cell was used to calibrate the intensity of the solar simulator to obtain a standard light intensity for characterization. Basic photovoltaic characteristics of the AIGS solar cells as a function of Na_2_Se amount are illustrated in Figure 13. Considering the systematic error, measurements were conducted on four different samples. Figure 13a–d shows the open-circuit voltage (*V_oc_*), short-circuit current (*J_sc_*), fill factor (*FF*), and conversion efficiency (*E_ff_*), respectively. The area of the solar cell was 0.2 cm^2^. As the thickness of Na_2_Se increased, all the basic parameters of *V_oc_*, *J_sc_*, *FF*, and *E_ff_* of the AIGS solar cell increased. The highest solar cell conversion efficiency of 6.6% (*V_oc_*: 775.6 mV, *J_sc_*: 15.5 mA/cm^2^, *FF*: 54.9%) was obtained when the thickness of the Na_2_Se layer was 20 nm. The basic characteristics of the other solar cells were as follows: *E_ff_* = 2.6% with *V_oc_* = 620.4 mV, *J_sc_* = 9.2 mA/cm^2^, and *FF* = 45.7% for 0 nm Na_2_Se; *Eff* = 3.6% with *V_oc_* = 662.1 mV, *J_sc_* = 11.8 mA/cm^2^, and *FF* = 46.1% for 5 nm Na_2_Se; *E_ff_* = 4.4% with *V_oc_* = 685.4 mV, *J_sc_* = 13.3 mA/cm^2^, and *FF* = 48.3% for 10 nm Na_2_Se.

**Figure 14:**
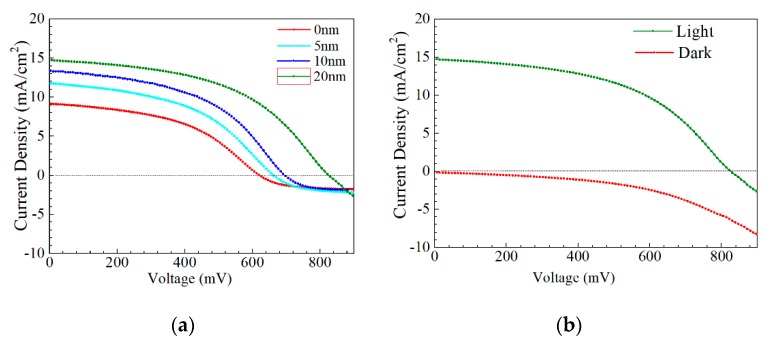

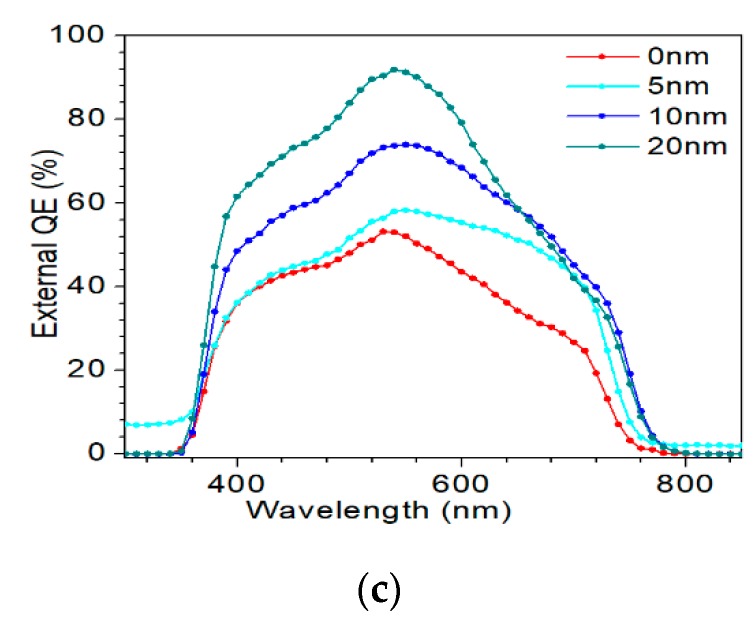
Performance of AIGS champion solar cells: (**a**) *J–V* curves as a function of Na_2_Se thickness; (**b**) light and dark *J–V* curve of AIGS solar cell with 20 nm Na_2_Se; (**c**) quantum efficiency as a function of Na_2_Se thickness.

*J–V* and *EQE* curves of the champion efficiency for the AIGS solar cells as a function of Na_2_Se thickness are shown in Figure 14a–c. In Figure 14a, when the thickness of Na_2_Se was <20 nm, an obvious roll-over in the high-voltage region was observed. This phenomenon can be attributed to two factors: (1) MoSe_2_ observed at the AIGS/Mo interface and (2) a high recombination rate because of low-quality solar cells. In this work, both factors are suggested to be influential, leading to the final result. Figure 14b shows the *J–V* curve of the AIGS solar cell with 20 Na_2_Se layers. No crossover was observed between the two curves. The *EQE* curve of the AIGS solar cell significantly improved with Na_2_Se thickness, as shown in Figure 13c. The *QE* curves show a rapid drop in the infrared region at ~750 nm, corresponding to the absorption edge of the AIGS film. Accordingly, the bandgaps of the AIGS films were calculated to be ~1.65 eV. Furthermore, the drops at ~510 and ~380 nm are attributed to the absorption edges of the CdS and ZnO layers [32], respectively. The correlation between *J_sc_* and the *EQE* curve is given as [33]

(1) $$J_{sc}=q\int_{0}^{\infty }QE\left(E\right)b_{s}\left(E,\;T_{s}\right)dE,$$

where *q* is the elementary charge and *b_s_* is the irradiation of the solar spectra. For standard conditions, air mass 1.5 (AM 1.5) is used, and *b_s_* is available from [34]. Based on Equation 1, Figure 13c, and the solar irradiation spectrum, *J_sc_* of the AIGS solar cell was calculated as 8.6 mA/cm^2^ for 0 nm, 10.6 mA/cm^2^ for 5 nm, 12.8 mA/cm^2^ for 10 nm, and 14.2 mA/cm^2^ for 20 nm. The deviation of *J_sc_* was calculated by the *QE* curve derived from the *J_sc_* obtained by the *J–V* curve, and is related to a lower illumination intensity of the QE measurement when compared to that of a sun-irradiated equivalent.

## 4. Conclusions

Ag_2_Se, In_2_Se_3_, and Ga_2_Se_3_ nanoparticle inks were fabricated via a wet ball-milling method, which, thereafter, were used to fabricate AIGS precursors via a spray method. The AIGS films were obtained by annealing the AIGS precursors under a Se atmosphere for 1 h at 570 °C. The influence of sodium doping on the AIGS solar cells was evaluated by vacuum-evaporating various thicknesses of Na_2_Se layers (0, 5, 10, and 20 nm) onto the Mo layer prior to fabricating the AIGS precursors. The grain size and crystallization of the AIGS films were significantly improved by sodium doping. Slight surficial sodium segregation and heavy sodium segregation at the AIGS/Mo interface were observed by SIMS. Basic photovoltaic characteristics of the AIGS solar cells were significantly improved as a function of increasing sodium content. The highest solar cell conversion efficiency of 6.6% (V_oc_: 775.6 mV, J_sc_: 15.5 mA/cm2, FF: 54.9%, with an area of 0.2 cm^2^) was obtained when the Na_2_Se thickness was 20 nm. The bandgaps of the AIGS thin films were calculated as 1.65 eV, according to the QE curve of the AIGS solar cell.
